# Middle Eastern mothers in Sweden, their experiences of the maternal health service and their partner's involvement

**DOI:** 10.1186/1742-4755-4-9

**Published:** 2007-10-24

**Authors:** Pernilla Ny, Lars Plantin, Elisabeth D Karlsson, Anna-Karin Dykes

**Affiliations:** 1 Dept. of Health Sciences, Lund University, Lund Sweden; 2 Faculty of Health and Society, Malmö University, Malmö Sweden

## Abstract

**Background:**

Traditional patterns relating to how to handle pregnancy and birth are often challenged due to migration. The purpose of this study was to describe Middle Eastern mothers' experiences of the maternal health care services in Sweden and the involvement of their male partner.

**Methods:**

Thirteen immigrant mothers from the Middle East who had used the maternal health services in Sweden were interviewed using focus group discussions and individual interviews. These were taped, transcribed and analysed according to Content analysis.

**Results:**

The four main categories that developed were:

• Access to the professional midwife

• Useful counselling

• Stable motherhood in transition

• Being a family living in a different culture

**Conclusion:**

According to the respondents in this study, understanding the woman's native language or her culture was not vital to develop a good relationship with the midwife. Instead the immigrant woman developed trust in the midwife based on the knowledge and the empathy the midwife imparted.

Increasing the amount of first trimester antenatal visits could avoid spontaneous visits to the emergency clinic. There was a greater need for involvement and support by the father during the perinatal period, such as caring for older children and carrying out household chores since the mothers' earlier female network was often lost.

**Clinical implications:**

There is a need to involve immigrant parents in the available parental education in order to prepare them for parenthood in their new country as well as to explore their altered family situation. Collecting immigrant women and their partner's, experiences of maternal health care services offers a possibility to improve the existing care, both in content, access and availability where the timing of visits and content require further evaluation.

## Background

Traditional patterns relating to how to handle pregnancy and birth are often challenged due to migration [1,2]. An earlier study showed how Middle Eastern men living in Sweden changed their traditional roles and took part to a greater extent in what was for them a 'woman's world' by giving support to the woman during pregnancy and childbirth [3]. The midwife has an important role in not only caring for and meeting the mother to be, but also in inviting the father to take part [4]. Several studies have emphasised the need for culturally congruent care, in the meaning that health professionals should be able to care for, and communicate with patients who belong to a different culture. [5]

Being born abroad with subsequent language difficulties can be perceived as a barrier to obtaining health care [6,7]. Fabian et al [8] could see that not speaking Swedish was related to non-attendance of parental education with some groups having adverse perinatal outcome [9,10]. More immigrant women underutilise the maternal health care [MHC] services than Swedish born women and they attend later for their first visit [11]. Involving their male partners during pregnancy and birth can enable the woman to receive support which is important both in transition to motherhood as well as in attitudes towards health preserving actions [12,13].

The Swedish maternal health care is carried out by midwives on a continuity basis at municipality clinics in accordance with the National recommendations. All parents are offered parental education, which is also provided by midwives. [4]

Immigrant women who are not fluent in the language of their adopted country are often excluded from research due to the lack of interpreters as well as an unawareness of the experiences of minority groups of immigrants. Including them offers a unique possibility to obtain their often different experiences [14]. Therefore the purpose of this study was to describe Middle Eastern mothers' experiences of the maternal health care services in Sweden and the involvement of their partners.

## Methods

### Setting

In Sweden antenatal care is offered free of charge. According to the National recommendations seven to nine visits are offered offered in accordance with the National recommendations and a further visit 8–12 weeks postpartum. [4] Those women whom the midwife considers to be at risk are refereed to a specialist antenatal clinic. One ultrasound scan performed by midwives is offered at 16–18 weeks with the purpose of determining the delivery date and detecting eventual foetal malformations. The present programme was introduced in 1996, recommending a reduced number of visits (earlier 13–14) as well as abolishing the earlier obligatory visit to a physician.

This study took place in Malmö, a multiethnic city in Southern Sweden with 270000 inhabitants of whom 26% were born abroad [15]. We used focus groups and individual interviews. Focus group A met at an antenatal clinic, group B at a university and group C at a school for immigrants. The first two individual interviews were conducted in the women's homes, two at a school for immigrants, and the remainder at an antenatal clinic.

### Participants

We invited 25 women to participate in the study and 13 agreed. Eight women accepted to participate in three different focus group discussions using Arabic language, and five other women accepted to be individually interviewed (one using an Arabic interpreter) in Swedish. After the postpartum check-up women were asked to take part by the interpreter, a member of the local Arabic speaking community, or by a midwife at an antenatal clinic in a multiethnic area or by a teacher working at a school for immigrants. At the antenatal clinic the women were asked by the midwife after conducting the postpartum check-up, if she would be interested in participating in this study. The researcher, using a female interpreter, explained the purpose of the study and asked again if the woman was interested in participating. If the woman understood Swedish she was then asked to participate in the individual interview. One week later the woman received a letter of invitation written either in Swedish or Arabic explaining the purpose of the study, the practical arrangements regarding time and place for the interview and their right to decline participation at any time. None of the participants was dependent on personal care from the researcher. Two of the women chose to be interviewed in their homes. In the case of the women who were asked to participate by their teacher, the whole class was first introduced to one of the researchers who explained the purpose of the study. Thereafter those women interested met the researcher individually according to a specific time schedule.

In order to be included in the study, immigrant women needed to be born in the Middle East [16] speaking Arabic as their mother tongue, had participated in the MHC in Sweden and were living or had lived together with a male partner from the Middle East.

The participants were women from Turkey, Syria, Iraq and Lebanon and most of them came to Sweden as refugees due to wars and conflict. The age varied from 23 to 41 years with their children ranging from 2.5 month to 21 years of age. The time lapse since participating in the Swedish MHC was between three months to eight years and the number of children per women was 1–6. Two of the women had given birth both in their native country and also in Sweden. The rest had participated in the Swedish MHC and given birth here. Their length of domicile in Sweden ranged from 4 to 19 years. The level of education was between six years of elementary school and up to university level, one woman was employed outside the home.

About half of the total group was living on social welfare, two women were divorced and eleven were married and living with a partner originating from the same area. Their ability to communicate in Swedish varied considerably.

Focus groups (A) and (B) included women who had lived in Sweden for 5 to 13 years, while the last group (C) consisted of women who had lived in Sweden for more than 18 years. Women having babies were represented in all three groups and all the women had participated in the Swedish MHC.

### Data collection

We used triangulation [17] with focus group discussions, individual interviews, interviews in the participants' native language and in Swedish in this exploratory research. Reading or writing either in Swedish or Arabic was not an inclusion criteria.

Conducting cross-cultural research adds layers of complexity and requiring the interpreter to translate as verbatim as possible [17].

We recorded all interviews and took extensive notes. The semi-structured interviews served as a guide that explored areas regarding the women's experience of the MHC and the participation of their male partner in the MHC. After consenting to participate in the study all participants filled in a short questionnaire regarding their socio-demographic data before participating in the interviews.

The research team, experienced in constructing questions for immigrants regarding antenatal care, developed the questions [3] and formed them into an interview guide. Using an interpreter, the guide was tested in one focus group interview with women born in Lebanon and now living in Sweden.

Each focus group discussion took 2 to 2.5 hours, and was always conducted by the same moderator and interpreter. The individual interviews took between 45 min and 1 h 20 min and were conducted in Swedish except one individual interview that was conducted in Arabic. The interviews were done in February to April 2005 and 2006.

Cultural research that crosses both cultural and language barriers needs special consideration. In this study we worked in close contact with an interpreter from the Middle East in order to minimise both linguistic and cultural misunderstandings.

The study was carried out with written informed consent from the respondents and according to the ethical principles of Biomedical Ethics. The study was conducted and approved in accordance with the Swedish legislation governing non invasive studies and the Helsinki declaration of 1996 [18].

### Data analysis

Content analysis is a suitable method for cross linguistic studies [19] and is used to determine what is significant [17]. The text was analysed according to the content analysis method by Burnard [20] and divided into codes, subcategories and categories. Key illustrative verbatim narratives reflecting the different sub-categories are presented, both as individual quotes and dialogues. First, the authors read the texts several times individually  to achieve an understanding of the different themes in the text. Thereafter, codes were attributed to the meaning units referring to the same content, therafter divided into subcategories and categories. The result of the content analysis was discussed with the interpreter to avoid misinterpretations of the content. Finally the authors agreed on the four main categories of the texts. In order to ensure consistency in the translation, parts of an audiotape from one interview were translated from Arabic into Swedish by an independent female translator from the Middle East with Arabic as her native language and who herself was not involved in the study.

## Findings

The following four main categories were developed in the analysis, presented with respective subcategories, Table I.

**Table 1:** The four main categories, presented with respective subcategories

**Main categories**	**Sub categories**		
**Access to the professional midwife**	To be respected and met with kindness	The trustworthy midwife who has the required knowledge	The need for frequent visits
**Useful counselling**	Counselling from the MHC	Parental group meetings, to receive correct information from a proper source	Information can also be frightening
**Stable motherhood in transition**	The mother is the best person for her child	Integration into Swedish society	
**Being a family in a different culture**	The presence of the husband	The female network	Experiences of the man as father and partner

Each main category is presented with its own sub-category/ies. We used quotes to illustrate both individuals and dialogues in the focus groups. (A-C describes the different focus groups interviews and 1–5 the individual interviews)

### Access to the professional midwife

#### To be respected and met with kindness

All women expressed that being met with kindness by someone who shows an interest in your situation is most important. Some women remarked on the approach of the staff in relation to questions as well as the individualised care. Women also appreciated the attention to detail which they had not experienced in their native country.

"I got more help during the first pregnancy (from the MHC) than now since I have more experience. When I need to I ask them and they answer me in a very nice way. Here (in Sweden) they take care of the woman and care for the little things.(3)

Some women had bad experiences with the care they had received in Sweden. One woman expressed a feeling of neglect, her midwife did not listen to her and did not take her worries seriously. Other women stated they had met midwives from their own country now working in Sweden and that they had been dissatisfied with their encounter. Even though the communication was simplified regarding language they wished that the attitude of these midwives towards them had been better.

*" That the staff knows our language is not enough, she has to be like you, helpful. To be kind is important. " *(C)

"I had a midwife originating from my home country, but she was not nice or helpful." (3)

None of the women expressed a wish for Arabic speaking staff, but several of them described the immense importance of being able to handle Swedish in their encounter with the midwife.

"It is so important to know the language so that the mother can tell herself, because the husband might not say exactly what she means." (4)

Having to talk via an interpreter regarding personal things such as obstetric and gynaecological matters was not satisfactory.

#### The trustworthy midwife who has the required knowledge

The midwife was considered trustworthy by the women on the basis of her knowledge and education. The same was expressed regarding the different physicians that the women had met. The difference in getting advice from a mother or a friend compared with the midwife is that the mother has only experience, but the midwife has both experience and professional training.

"I listen to the midwives advice; my mother is not a midwife or a physician. The midwife is educated so she knows more." (5)

The midwife knows what she is doing because she has studied and has experience so I have to listen to her. If I have a question I ask her. I do trust the midwives and the physicians. " (1)

Some of the women also stated that they felt safe because the MHC takes care of both their health and the baby's.

"You feel safe when you go to the MHC, they think about everything and they take care of you and your child. You don't have to be afraid of unhygienic conditions or to get infected by diseases." (2)

#### The need for frequent visits

Several of the women stated that there was a long time lapse from their first visit to the MHC in early pregnancy to the second.

This is a time of both physical and emotional change for the woman and her partner, and a need for communication with the midwife. If this need is not satisfied some women said that they might feel the need to seek care elsewhere.

One of the groups noted in their discussions:

"-I wonder if there is a possibility to have an extra visit between the first and the second? During this time there are so many changes in the woman's body. It is important for both body and mind that a pregnant woman gets support and information from a knowledgeable person. (Participant 1)

- A woman needs a lot of information during this first period, not later when all has calmed down. (Participant 2)

- Otherwise you have to seek care at the emergency clinic and there it is difficult to get through and get help (Participant 1)

- An appointment to a midwife can give a feeling of security and help women not to get anxiety or depressed. During this time a pregnant woman needs someone to talk to who is patient and keen." (Participant 1) (A)

### Useful counselling

#### Counselling from the MHC

Women declared that information, advice and the possibility to discuss their own health situation and the status of their unborn child was very important. One woman stated that the midwife both gave advice and listened, but left the decision making up to her.

"The midwife tried to comfort me and she gave advice. But I didn't need to follow her advice, even though she listened she did not force me to do anything, they just give you information I mean."(A)

The MHC seemed important to the women and one woman said (C) *" Nothing can stop the woman from going to MCH, nothing is more important. The child can die otherwise!"*

#### Parental group meetings, to receive correct information from a proper source

Receiving information as a group was considered positive among all women, but not all women said they needed information since they had friends and family with whom they could talk. Also the women's opinions about the participation of their partners differed. Some women thought if their male partner was present, they could not talk about breastfeeding or other more intimate matters. On the other hand, other women said that these things were natural and nothing to be ashamed of:

"-I went there because I got information at the right time. They were sort of one step ahead, so one knew what to expect. (Participant 1)

-I did not feel a need, but I was invited. I have family and also friends that have children already, so they know a lot. (Participant 3)

-I went there with my husband, but he only came twice since he got bored. He does not understand Swedish that well. It was hard to go by your self because every one else was there as a couple." (B) (Participant 1)

#### Information can also be frightening

In some societies talking about risks may be unacceptable due to the belief this makes bad things happen. Some women believed that the outcome of pregnancy is not for them to decide, it is either in Gods hands or in the hand of the fate. Women also stated they wanted information but at the same time it could also be frightening:

"-I think it is good with information so that you can be prepared if something happens. (Participant 1)

-Yes, but they should only say things they are certain about otherwise you get really worried. (Participant 2)

-Yes, because if it then turns out that there wasn't anything wrong you can't trust the person again. How could she have been so wrong?" (Participant 3) (B)

When the women were asked if they thought it was worth getting information that is frightening they all stated that it was worth it as the advantages predominated.

### Stable motherhood in transition

#### The mother is the best person for her child

Nearly all women stated that the mother is the best and most central person for their children. The mother is the one who takes care of all the practical things, but also the one to whom the children want to talk to. Her role is to be responsible for the children's upbringing and their progress in school as she has more patience than the father and is more committed to her children.

Being in a transition caused several of the women to make comparisons between motherhood in their native country and in Sweden. According to the participants the ability to be a mother was something women had in them: *"-Yes women are born with it, it comes natural for her."(B) *and it is stable. In the move to a new country the external conditions could be different, such as division of labour within the family and different housing conditions:

"In Iraq the men work fulltime and the women part time so women would have had more time there for her children there. We would also have lived in a big house. The society affects the conditions! " (B)

#### Integrating into Swedish society

Three of the mothers expressed their wish to go to work, feeling unsatisfied to be dependent on social allowance. Some women had not been able to go to school regularly affecting their ability to learn Swedish and to find a job here.

"-Due to war in the homeland I could not study since the school was closed. I don't have an education so it is hard to get a job here."(4)

"If the woman has a job and feel well she can raise her children in a better way. If the mother is not well the children does not feel well either."(4)

Most of the woman did not have any close friends among their fellow countrymen either.

### Being a family in a different culture

#### The presence of the husband

All women, except one, described the presence of their husbands both at the MHC and during the delivery in a positive way. Some men had been actively involved in conversations with the midwife and some had stated that they were there as support only because it concerned the woman's body. Being able to utilise the MHC services by herself made the woman feel less dependent on her husband. According to the women their partners expressed a need for the woman to be independent.

"No, the midwife only talked to me. He wanted it that way since this is my thing and it is important that I know what is happening. If I need help I know he will help me."(1)

"He said you have to be independent to be strong."(5)

Nearly all women said that giving their husband the chance to take part in what was formerly considered to be a 'woman's world' gave him the possibility to see her efforts during delivery and a chance to get an earlier and closer contact with his child.

"The presence of the man is needed to share the woman's suffering. Sometimes you just have to get him in the picture. "(A)

#### The female network

The mother has experience of pregnancy and childbirth and would normally be a support during her daughter's confinement. Due to problems obtaining entry permits into Sweden not all the women's mothers living outside Sweden could visit their daughters during the perinatal period.*"It is only ones mother that can help. You can not expect that much from the husband. He soon gets bored and gives up the new responsibility."(A)*

"If I could make a wish I would like to have my mother present at the delivery."(5)

Many women noted that since coming to live in Sweden they had no relatives or family except for their husband, which made raising the children more difficult.

"It is a heavy responsibility in Sweden because in the home country there is always someone that helps, such as a mother in law, an aunt or sister. Everyone plays their part in the care and upbringing of the child."(A)

"I had no one to ask when I was pregnant for the phone calls to my mother were really expensive and you cannot handle the cost when you live on subsidies from the government. Sometimes I asked my friends, but I don't know that many so life was difficult."(3)

#### Experiences of the man as a father and partner

Life in Sweden was often different compared to life in the native country. Below is a dialogue concerning men within a family living in Sweden:

"Difficult experiences due to war is the reason for the lethargy among our men. Those fathers living in Sweden who mostly stay in the home could be disturbing for the women. (Participant 1)

-The life, the system and the culture is very different in Sweden compared to that of our home countries. (Participant 3)

-Here in Sweden women's development runs parallel to that of the men. She goes to school, learns the language and receives just as many hours at school as the man. This creates a problem for the men who have the traditions and praxis that the woman should take care of the household by herself. This, the man does not want to loose." Participant 2) (A)

Some women also expressed a wish to be able to continue taking the main responsibility for the household.

"When the mother cooks and cleans at home and the father goes to work the children feel that they have a family." (2)

Among those women who did not work, all of them expressed concern as to how they should be able to take care of their family and go to work at the same time. They also stated that it is difficult, nearly impossible, to live on a single salary.

Women expressed when living in Sweden there is a need for mutual understanding within the family concerning division of labour, since couples are living on their own without the larger family circle common in their countries of origin. Some of the women could also see this new situation, without the involvement of the extended family, as being positive.

"Here in Sweden the mother is strong and she can decide. In our home-country you have to listen to the rest of the family. It is better for me here. Life with my husband is also different. We both decide what is best for our children so our life is better."(1)

In focus group (B) the women discussed how the different circumstances in the country one is living affects you:

"-In the home country the husband works more and the woman less so she has time for her children. In Sweden I always have a bad conscience since I am working so much, but that depends on me not having been raised there. If I had been raised in Sweden I would not have thought about it. Now since my husband has time he mostly takes care of our child. That is good, because now he is a real father, not just a father on paper.(Participant 1)

-Yes it is the society you live in that forms you. In Sweden there are other conditions." (Participant 2) (A)

## Discussion

Trustworthiness is central in qualitative research and includes concepts such as stability and credibility. The authors have analysed the data separately in order to assure that the descriptive categories are in accordance with the interview material. The text was read several times in order to achieve stability [21]. The discussions among the authors reduced the risk of researcher bias and enhanced the validity; the risk of the latter was also minimised by using triangulation – a combination of methods [17]. To maximize the quality of the data it is important to interview participants in their native language [22] by using the same interpreter for each interview [19].

Based on the experience from previous research [22] our focus groups consisted of 2–4 participants. By using small groups we eliminated the risk of any frustration caused to the participants by not having enough time or the possibility to fully express themselves. We followed the concept of 'higher involvement' where the participants were seen as being the experts [23].

Twelve women could not participate due to personal reasons, after first having accepted to be part of the study. Difficulties in recruitment when conducting research among immigrant groups in Australia are described by Small et al [24]. This can reflect on the validity of the results [25] and must be considered when interpreting them.

We used several focus groups and individual interviews to increase the validity of our results [25,26]. The reason for not using the same informants in focus groups and individual interviews was the desire for variety as proposed by Twinn [19], since finding common patterns in a heterogeneous group of people is of particular interest [17].

Conducting interviews in the participants native language seems to be important. Even though the participants in the individual interviews had lived in Sweden for several years their ability to talk about private matters in Swedish was limited. It was not always possible for the participants to fully express their true meaning, which has to be taken in consideration when interpreting the results [27].

Women taking part in this study have experienced seeing other women suffer during pregnancy and childbirth in their home countries, often related to war and economical restraints. In Sweden, care is given by professional caregivers with large medical resources and is free of charge. Women who participated in this study were grateful for the maternal health care offered to them and the way it is provided. This was similar to the findings of a study of immigrant women from the Middle East living in Australia [28].

### Trust in the MHC

In this study, in contrast to others [29-32] none of the women expressed a need for a midwife to be knowledgeable about their culture or able to speak their native language. An approach by a knowledgeable and empathetic midwife was experienced as trust promoting. Deveugele et al [33] state that good communication requires both knowledge and empathy where the interaction between patient and care provider is most important [34]. Furthermore being met with kindness and a feeling of acceptance was vital to the health of immigrant families in Sweden [35]. "*Culturally competent care*" entails several properties which stress the importance of recognising differences as well as creating trusting relationships [36], emphasizing on the importance to see beyond the context of religion, cultural and ethnic background and focus on the individual woman[28].

The time lapse between the first and second visit (gestational week 12 to 25) in the antenatal care programme was felt as being too long and could increase the number of emergency visits. The first trimester is the time when most changes affect the woman's body, both physically and psychologically. This could be a period where increased contact with antenatal care personnel may be of benefit. The National recommendations in Sweden [4] are in the process of change, both in content and in regard to the number of visits.

According to the women in this study, information and communication are central issues, but as shown in Nigenda et al [37] this can differ according to cultural background. The women in our study noted that midwives give advice, but leave it to the individual woman to make her own decision; a different experience than the feeling of being pressured into action, [28] or a feeling of ignorance [31]. The Swedish National Board of Health and Welfare [4] provides recommendations regarding antenatal care, but it is the responsibility of the midwife to implement the care individually and in agreement with the pregnant woman.

### Parenthood in transition

By transition the authors mean the effects of migration and how immigration can influence parenthood [38]. Women saw their male partners as supporters, someone to pave the way for them during pregnancy and delivery. Some women expressed that being supported by their partners is essential to their effort to manage themselves in this new society, both in their contacts with health staff and in daily life. The women welcomed this idea similar to the findings of Wiklund et al [39]. The Middle Eastern societies mostly have a patriarchal structure and moving to Sweden can create conflicts due to different ways of looking at gender both in society in general and also within family life [40-42].

The women in this study had immigrated for different reasons, they had moved from a traditional family/group oriented society to a Western individualised society [41] Although life with their partners and life as a woman was in transition, the feeling for motherhood was stable. This is opposite to the experiences of immigrant men from the Middle East in Sweden [3]. Men experienced an altered balance of power within the family when living in Sweden and a change in their position as a father. They still saw themselves as the providers, but since they were unemployed they could not support their family economically. In this situation, immigrant men can see their role as a leader and decision maker threatened [3,40]. Fatherhood seems to be more dependent on external factors and is more easily affected by external dynamics than motherhood. Some women regarded their husbands unsupportive since they quickly became less enthusiastic about their new position as a father. Meadows et al [43] found that many immigrant women in Western societies feel isolated in an alien system, devoid of their female relatives. Women felt that if men would be more involved in daily activities around their children they would not only become emotionally more close to their children, but would also gain a better understanding of women's work at home. Women in our study had experienced that change in situation affects their family life. Some even had a total opposite view of partnership and fatherhood than the immigrant men when living in Sweden. A father taking practical care of his children was a real father, *"not a father only on paper" *(B). Women spoke about how a couple should help and support one another both in family matters and in matters related to society. This was also referred to by some men realising that since the Swedish society is different "*I, as a man have to change otherwise the family will not last *". [3] Men who were employed expressed that they had finally found their place as full citizens since they contributed to society and paid taxes. That gave them the confidence to also view family life and gender related issues differently.

## Conclusion

Language differences did not seem to influence the quality of antenatal care. In order to develop a trusting relationship, knowledge and empathy of the attending midwife were important factors. The need for more visits in the first trimester seemed to be important, not only for the individual woman but also as an attempt to avoid spontaneous visits to the emergency clinic.

Due to migration the relationship within the family had altered; there was a need for involvement and support by the husband in the perinatal period, since the mother's earlier female network was often lost. According to the women, not all men managed to give this support which often placed the woman in a stressful situation at the time after the birth.

## Clinical implications

There is a need to involve immigrant parents in parental education in order to prepare them for the reality of parenthood in their new country as well as for exploring their changed family situation. Collecting immigrant women and their partner's experiences of the MHC, offers a possibility to improve the existing care, both in access and availability, where the timing of visits and content require further evaluation.
